# Estimating the productivity of radiologists in Brazil: the search for a benchmark

**DOI:** 10.1590/0100-3984.2019.0081

**Published:** 2020

**Authors:** Elisa Almeida Sathler Bretas, Ruy Moraes Machado Guimarães, André Yui Aihara, Hilton Muniz Leão Filho, Rubens Chojniak, Valdair Francisco Muglia, Giuseppe D’Ippolito

**Affiliations:** 1 Grupo Fleury, São Paulo, SP, Brazil.; 2 Centro Médico de Campinas, Campinas, SP, Brazil.; 3 Escola Paulista de Medicina da Universidade Federal de São Paulo (EPM-Unifesp), São Paulo, SP, Brazil.; 4 Instituto de Radiologia do Hospital das Clínicas da Faculdade de Medicina da Universidade de São Paulo (InRad/HC-FMUSP), São Paulo, SP, Brazil.; 5 A.C. Camargo Câncer Center, São Paulo, SP, Brazil.; 6 Faculdade de Medicina de Ribeirão Preto da Universidade de São Paulo (FMRP-USP), Ribeirão Preto, SP, Brazil.

**Keywords:** Radiologists/standards, Efficiency, Remuneration, Reimbursement, incentive, Workload, Diagnostic imaging/statistics & numerical data, Radiologistas/normas, Eficiência, Remuneração, Reembolso de incentivo, Carga de trabalho, Diagnóstico por imagem/estatística e dados numéricos

## Abstract

**Objective:**

To determine the average productivity of radiologists, as measured by number of reports issued per 6-h shift, evaluating variables that could affect the results.

**Materials and Methods:**

This was a study utilizing an online questionnaire sent to radiologists affiliated with the Brazilian College of Radiology and Diagnostic Imaging. The questions were related to the demographic profile and professional practice characteristics (form of remuneration, primary imaging method employed, and subspecialty) of the radiologists, as well as their individual productivity (average personal productivity) and the productivity considered reasonable in a 6-h shift. The association between productivity and the practice characteristics of the radiologists was determined by using Poisson regression to calculate the prevalence ratio.

**Results:**

A total of 510 radiologists completed the questionnaire. The great majority of the respondents (84%) reported that their remuneration is directly related to their productivity. The productivity varied according to the subspecialty, work environment, and remuneration model.

**Conclusion:**

We demonstrated that the productivity of radiologists is associated with the characteristics of their employment. We hope that this study will encourage other studies aimed at evaluating the productive capacity of the radiologists in Brazil, addressing the various functions they perform in their daily routine, including activities other than issuing reports.

## Abstract (pt)

**Objetivo:**

Estimar a produtividade média dos radiologistas brasileiros em número de laudos emitidos por período de trabalho de seis horas, analisando variáveis que possam influenciar os resultados.

**Materiais e Métodos:**

Pesquisa realizada por meio de questionários *online* respondidos por radiologistas brasileiros afiliados ao Colégio Brasileiro de Radiologia e Diagnóstico por Imagem. As questões incluíram dados demográficos e profissionais dos radiologistas (forma de remuneração, método de imagem de atuação e subespecialidade) e a produtividade individual e a considerada razoável em um período de seis horas de trabalho. A associação entre a produtividade e as características de trabalho dos radiologistas foi calculada pela razão de prevalência, por meio da regressão de Poisson.

**Resultados:**

Ao todo, 510 radiologistas responderam ao questionário. A grande maioria dos respondedores (84%) relatou que a sua remuneração está diretamente relacionada à produtividade. A produtividade variou em função da subespecialidade de atuação, ambiente de trabalho e modelo de remuneração.

**Conclusão:**

Demonstramos a associação entre a produtividade do radiologista e as características relacionadas à forma de trabalho. Esperamos que este estudo impulsione outras pesquisas que avaliem a capacidade produtiva do radiologista brasileiro, considerando as diversas funções exercidas por este profissional em sua rotina de trabalho, contemplando outras atividades, além da emissão de laudos.

## INTRODUCTION

Measuring the productivity of radiologists has been the focus of growing interest from professional associations, employers, and even service providers themselves. This information is essential to understanding how professionals have been adapting to changes in the job market, largely determined by the growing demand for imaging examinations and, above all, by the introduction of new technologies. In this context, several processes designed to measure and manage the productive capacity of radiologists have been developed and studied, the aim being to increase efficiency without impairing the quality of the service provided to patients or the job satisfaction and work environment of the radiologists^(1-3)^.

Studies conducted in the United States, Europe, and Australia have shown an increase in the workload and productivity of radiologists in recent decades^(1,4,5)^. A recent survey conducted by the Colégio Brasileiro de Radiologia e Diagnóstico por Imagem (CBR)-Brazilian College of Radiology and Diagnostic Imaging-showed that the majority (approximately 62%) of radiologists in Brazil work 8-12 h per day and that approximately 7% work > 12 h per day^(6)^.

Among the many possible ways to measure the productivity of radiologists is tracking the number of reports issued over a certain period of time. However, estimating the “true productivity” of these professionals is a great challenge, because their work encompasses at least four aspects: pre-examination assessment of the case to evaluate the appropriateness of the study; imaging quality check; image interpretation; and preparation of reports/providing consultations to patients or referring physicians^(5)^. In addition, the daily routine of radiologists includes other activities that cannot be easily quantified, such as participation in multidisciplinary meetings, teaching, research, and administrative tasks^(4)^. We also believe that there are differences in work environment, remuneration model, examination complexity, location, and subspecialty that can affect the number of reports issued.

The purpose of this study was to estimate the average productivity of radiologists in Brazil during a 6-h shift. To that end, we analyzed the completed questionnaires received from members of the CBR and evaluated variables that could influence the results.

## MATERIALS AND METHODS

This was a cross-sectional study conducted in the second half of 2018. The data were obtained through online questionnaires (Google Forms) sent by email to 5600 CBR-registered radiologists. The questionnaire was prepared and validated by radiologists who are members of the CBR report committee. All responses were optional and anonymous (Appendix I). The survey was closed 12 weeks after the email messages were sent. According to the rules for the protection of human subjects in research, studies that are based on anonymous and optional questionnaires do not require approval by a research ethics committee^(7)^.

The questionnaire was divided into two parts. The first part contained questions about the demographic profile of the radiologists, including variables such as age; region of origin; whether they are based in the capital city or in the countryside of their state; predominant work environment-public or private hospital, private clinic, or teleradiology service; predominant remuneration model-fixed pay per shift, productivity-based, or mixed; predominant imaging method employed-X-ray, ultrasound, computed tomography (CT), magnetic resonance imaging (MRI), or other; and subspecialty-general radiology, abdominal radiology, musculoskeletal radiology, neuroradiology, or thoracic radiology). The second part of the questionnaire contained questions related to their perceived individual productivity (mean personal productivity) and expected productivity (minimum and maximum number of reports considered reasonable) over a 6-h shift.

Null data (i.e., those related to questions that were left unanswered) were excluded from the statistical analysis. We used the 2016 version of Excel for Windows to build a database for the statistical analysis and to determine the frequency distribution of all variables analyzed in the study. The following descriptive statistics were calculated for numerical variables: mean, median, standard deviation, standard error, and interquartile range. We used Poisson regression^(8)^ to calculate the prevalence ratio (PR) and determine whether there was any association between dependent variables and certain characteristics/working styles of the radiologists.

## RESULTS

### Descriptive analysis

As can be seen in Table 1, a total of 510 radiologists working in Brazil completed the questionnaire (response rate = 9.1%). Of those, 53% were between 30 and 39 years of age, 53% lived in the southeastern region of the country, and 67% worked in a capital city. The main imaging method employed was ultrasound in 38%, CT in 29%, MRI in 26%, X-ray in 16%, and other in 2%. General radiologists accounted for 59% of the respondents, and the most common subspecialties were internal medicine (abdominal, pelvic, and thoracic radiology), in 23%, musculoskeletal radiology, in 10%, and neuroradiology (7%), CT and MRI being the prevailing examinations performed by the subspecialists.

**Table 1:** Average personal productivity (number of reports per 6-h shift), by respondent characteristic.

Characteristic	Average personal number of reports						
	Number of responses	Mean	Standard deviation	Standard error	Median	25th percentile	75th percentile
Age							
20-29	26	35.8	65.0	12.7	20.0	15.0	30.0
30-39	271	23.3	10.3	0.6	20.0	16.0	30.0
40-49	103	27.6	17.2	1.7	25.0	18.0	30.0
50-59	77	28.2	40.2	4.6	22.0	18.0	30.0
> 60	33	34.8	38.0	6.6	20.0	15.0	35.0
Total	510	26.2	25.8	1,4	20.0	17.0	30.0
Region of Brazil							
Central-west	47	22.2	8.3	1.2	20.0	15.0	25.0
North-northwestern	112	25.2	12.8	1.2	20.0	17.0	30.0
Southwestern	271	28.4	32.0	1.9	22.0	18.0	30.0
South	80	22.8	22.0	2.5	20.0	14.0	28.5
Locality							
State capita l	341	27.1	28.8	1.6	22.0	17.0	30.0
Elsewhere	169	24.5	18.4	1.4	20.0	16.0	28.0
Work environment							
Private clinic	247	26.2	27.3	1.7	20.0	18.0	30.0
Private hospital	173	27.0	28.3	2.2	20.0	16.0	30.0
Public hospital	72	24.0	14.4	1.7	20.0	14.0	30.0
Teleradiology	18	29.4	15.2	3.6	25.0	22.0	30.0
Remuneration model							
Mixed	114	25.5	15.0	1.4	22.0	16.0	30.0
Per-shift	79	24.9	23.9	2.7	20.0	15.0	28.0
Productivity-based	317	26.9	29.2	1.6	22.0	18.0	30.0
Imaging method							
X-ray	16	83.9	109.0	27.3	42.5	27.5	85.0
MRI	137	21.0	12.6	1.1	20.0	15.0	24.0
CT	150	25.3	11.5	0.9	23.0	20.0	30.0
Ultrasound	198	25.8	16.4	1.2	22.0	18.0	30.0
Other	9	29.4	30.6	10.2	20.0	5.0	40.0
Subspecialty							
Abdominal pelvic radiology	106	23.1	9.8	0.9	20.0	16.0	29.5
General radiology	301	28.7	31.9	1.8	23.0	18.0	30.0
Musculoskeletal radiology	50	20.8	6.5	0.9	20.0	15.0	24.8
Neuroradiology	38	25.3	21.3	3.5	20.0	14.2	27.2
Thoracic radiology	15	20.9	9.8	2.5	20.0	15.0	26.5

The majority of the respondents (83%) worked predominantly at private facilities, 48% working at clinics and 33% working at hospitals. Only 15% of the respondents worked predominantly at public hospitals, and only 3% worked for teleradiology services (public or private). The majority of the respondents (84%) stated that their productivity is taken into account for the calculation of their remuneration, 62% receiving pay that was based only on the number of reports issued and 22% working within a remuneration model with a fixed component and a variable component, the latter depending on their productivity. Only 15% of the respondents worked primarily within a remuneration model with a fixed pay per shift (Table 1).

Tables 1, 2, and 3 list, respectively, the average personal productivity, the minimum productivity per 6-h shift considered acceptable, and the maximum productivity per 6-h shift considered acceptable, within the context of the daily practice of the respondents. The subsequent analysis takes into account the medians calculated so as to prevent outliers from influencing the results. Overall, the self-reported median number of examinations performed per 6-h shift (20) is within the expected minimum and maximum acceptable limits defined by the respondents (16 and 28, respectively). For those reporting predominantly X-ray examinations, the minimum and maximum numbers of reports considered acceptable were 40 and 50, respectively. For those reporting predominantly ultrasound examinations, the minimum and maximum numbers of reports considered acceptable were 18 and 30, respectively. Radiologists who worked predominantly with CT and MRI were divided by subspecialty. Abdominal examinations were subdivided into examinations of the upper abdomen and examinations of pelvis. General radiologists considered 18 and 30 to be the acceptable minimum and maximum number of reports per 6-h shift, respectively, whereas those limits were defined as 15 and 30, respectively, by abdominal radiologists, 15 and 27, respectively, by neuroradiologists, 15 and 25, respectively, by musculoskeletal radiologists, and 18 and 25, respectively, by thoracic radiologists.

**Table 2:** Minimum number of reports per 6-h shift considered acceptable, by respondent characteristic.

Characteristic	Minimum number of reports						
	Number of responses	Mean	Standard deviation	Standard error	Median	25th percentile	75th percentile
Age							
20-29	26	20.1	21.5	4.2	15.0	10.0	20.0
30-39	271	17.3	7.7	0.5	15.0	12.0	20.0
40-49	103	18.8	10.6	1.0	18.0	11.0	24.0
50-59	77	19.1	14.2	1.6	18.0	12.0	21.0
> 60	33	20.6	13.1	2.3	15.0	12.0	20.0
Total	510	18.2	10.8	0.5	16.1	12.0	20.0
Region of Brazil							
Central-west	47	16.7	6.5	0.9	15.0	12.0	20.0
North-northeastern	112	17.4	9.4	0.9	15.0	10.0	20.0
Southwestern	271	19.7	12.7	0.8	18.0	12.0	20.0
South	80	15.5	7.1	0.8	15.0	10.0	20.0
Locality							
State capital	341	18.8	12.0	0.6	15.0	12.0	20.0
Elsewhere	169	17.1	8.2	0.6	15.0	12.0	20.0
Work environment							
Private clinic	247	18.4	10.4	0.7	15.0	12.0	20.0
Private hospital	173	18.4	11.8	0.9	15.0	12.0	20.0
Public hospital	72	16.6	10.2	1.2	15.0	10.0	20.0
Teleradiology	18	20.7	11.6	2.7	20.0	15.8	20.0
Remuneration model							
Mixed	114	17.7	9.8	0.9	15.0	10.0	20.0
Per-shift	79	16.3	8.1	0.9	15.0	12.0	20.0
Productivity-based	317	18.9	11.8	0.7	15.0	12.0	20.0
Imaging method							
X-ray	16	43.7	34.8	8.7	40.0	15.0	50.0
MRI	137	14.9	7.2	0.6	15.0	10.0	18.0
CT	150	18.3	8.2	0.7	18.0	12.0	20.0
Ultrasound	198	18.8	8.3	0.6	18.0	12.0	24.0
Other	9	13.6	12.0	4.0	12.0	3.0	20.0
Subspecialty							
Abdominal pelvic radiology	106	17.5	7.3	0.7	15.0	12.0	20.0
General radiology	301	19.2	12.6	0.7	18.0	12.0	24.0
Musculoskeletal radiology	50	15.4	6.0	0.9	15.0	10.0	19.5
Neuroradiology	38	17.4	10.9	1.8	15.0	10.0	20.0
Thoracic radiology	15	16.0	6.4	1.7	18.0	12.0	20.0

**Table 3:** Maximum number of reports per 6-h shift considered acceptable, by respondent characteristic.

Characteristic	Maximum number of reports						
	Number of responses	Mean	Standard deviation	Standard error	Median	25th percentile	75th percentile
Age							
20-29	26	33.3	27.9	5.5	29.0	20.0	40.0
30-39	271	29.2	12.8	0.8	26.0	20.0	35.0
40-49	103	32.8	22.6	2.2	30.0	20.0	40.0
50-59	77	31.4	33.0	3.8	25.0	20.0	30.0
> 60	33	34.7	21.6	3.8	25.0	20.0	48.0
Total	510	30.8	20.6	0.5	28.0	20.0	36.0
Region of Brazil							
Central-west	47	29.0	11.2	1.6	25.0	20.0	35.5
North-northeastern	112	29.1	13.8	1.3	25.0	20.0	37.5
Southwestern	271	33.2	25.6	1.6	30.0	20.0	36.0
South	80	26.5	11.5	1.3	24.0	18.0	30.0
Locality							
State capita l	341	32.0	23.8	1.3	30.0	20.0	40.0
Elsewhere	169	28.5	11.9	0.9	25.0	20.0	35.0
Work environment							
Private clinic	247	30.7	22.8	1.5	27.0	20.0	35.0
Private hospital	173	31.5	19.7	1.5	30.0	20.0	40.0
Public hospital	72	28.2	14.9	1.8	24.5	16.0	40.0
Teleradiology	18	37.1	18.2	4.3	32.5	25.8	40.0
Remuneration model							
Mixed	114	30.3	15.1	1.4	29.0	20.0	39.0
Per-shift	79	28.5	19.6	2.2	24.0	20.0	30.0
Productivity-based	317	31.6	22.5	1.3	30.0	20.0	36.0
Imaging method							
X-ray	16	80.7	75.3	18.8	50.0	35.0	100.0
MRI	137	27.4	17.2	1.5	25.0	20.0	30.0
CT	150	31.1	13.8	1.1	30.0	20.0	40.0
Ultrasound	198	29.3	11.1	0.8	30.0	20.0	35.0
Other	9	28.3	22.4	7.5	20.0	12.0	50.0
Subspecialty							
Abdominal pelvic radiology	106	29.9	12.7	1.2	30.0	20.0	40.0
General radiology	301	31.6	23.2	1.3	30.0	20.0	36.0
Musculoskeletal radiology	50	27.1	9.8	1.4	25.0	20.0	35.0
Neuroradiology	38	33.8	28.2	4.6	27.5	20.0	39.0
Thoracic radiology	15	27.9	14.3	3.7	25.0	18.0	40,0

### Multivariate analysis

Considering the medians found, we found that productivity was associated with independent variables such as region, locality (state capital or other), work environment, remuneration model, predominant imaging method employed, and subspecialty (Table 4).

**Table 4:** Multivariate analysis of radiologist productivity in Brazil.

Characteristic	PR (95% Cl)
Age	
20-29	1.0 (reference)
30-39	0.65 (0.61-0.70)
40-49	0.77 (0.72-0.83)
50-59	0.79 (0.73-0.85)
>60	0.97 (0.89-1.06)
Region of Brazil	
Central-west	1.0 (reference)
North-northeastem	1.14 (1.06-1.22)
Southwestern	1.28 (1.20-1.37)
South	1.03 (0.95-1.11)
Locality	
State capital	1.0 (reference)
Elsewhere	0.90 (0.87-0.94)
Work environment	
Private clinic	1.0 (reference)
Private hospital	1.03 (0.99-1.07)
Public hospital	0.91 (0.87-0.96)
Teleradiology	1.12 (1.03-1.23)
Remuneration model	
Mixed	1.0 (reference)
Per-shift	0.98 (0.92-1,03)
Productivity-based	1.06 (1.00-1.10)
Imaging method	
Other	1.0 (reference)
X-ray	2.85 (2.50-3.25)
MRI	0.71 (0.63-0.81)
CT	0.86 (0.76-0.97)
Ultrasound	0.88 (0.77-0.99)
Subspecialty	
Abdominal pelvic radiology	1.0 (reference)
General radiology	1.24 (1.19-1.30)
Musculoskeletal radiology	0.90 (0.84-0.97)
Neuroradiology	1.10 (1.02-1.18)
Thoracic radiology	0.90 (0.80-1.02)

95% CI, 95% confidence interval.

We found an association between productivity and the region of the country where the radiologists worked. Taking the radiologists in the central-west region as the reference, we found that productivity was 28% higher in the southeastern region (PR: 1.28); 14% higher in the north-northeastern region (PR: 1.14); and 3% higher in the southern region (PR: 1.03). Living in a state capital was another factor associated with productivity. The productivity reported by radiologists based in state capitals was 10% higher than that reported by those based elsewhere (PR: 1.10).

We also identified an association between work environment and productivity. Taking the productivity of radiologists working predominantly at private clinics as the reference, we found that the productivity of those working in teleradiology was 12% higher (PR: 1.12) and that of those working at private hospitals was 3% higher (PR: 1.03), whereas that of those working at public hospitals was 10% lower (PR: 0.90).

Among our respondents, there was a clear association between productivity and the model of remuneration. Taking the productivity of radiologists who get paid through a mixed remuneration model as the reference, we found that the productivity of those whose remuneration was solely dependent on the number of reports issued was 6% higher (PR: 1.06), whereas that of professionals who received a fixed amount per shift was 2% lower (PR: 0.98).

The productivity reported by general radiologists was higher than that reported by those working in subspecialties. Taking the productivity of abdominal radiologists as the reference, we found that the productivity of general radiologists was 24% higher (PR: 1.24) and that of neuroradiologists was 10% higher (PR: 1.10), whereas that of musculoskeletal and thoracic radiologists was 10% lower (PR: 0.90).

## DISCUSSION

Our study showed that, according to the opinion of the respondents in our study sample, the productive capacity of radiologists, as measured by the number of reports issued per 6-h shift, varies depending on subspecialty, work environment, and remuneration model, among other factors.

In general, the median number of reports considered the acceptable minimum was 15-18 and that considered the acceptable maximum-a number that, to ensure the well-being of radiologists and the high quality of their work, should not be exceeded-was 25-30, and those values were relatively homogeneous. The literature, however, is quite inconsistent when it comes to that. There is evidence that the mean time for CT and MRI report writing ranges from 40 to 110 min for patients seen in the emergency room by radiologists with one or two years of experience after their third year of residency^(9)^. That would translate to a productivity of no more than 6-12 reports per 6-h shift. In contrast, other studies have reported that that the mean time for writing the report of a CT examination of the abdomen and pelvis is 10-15 min, which would translate to a productivity of 24-36 reports per 6-h shift^(10)^.

It is noteworthy that the recommended or suggested productivity target is somewhere in between the minimum and maximum values reported in our study, given that these numbers would apply to the working hours in which radiologists are solely dedicated to image reading, and not involved in other activities that naturally make up their routine^(4)^, such as patient care; case discussion with referring physicians (medical consultation); interventionist procedures; treatment of complications; participation in administrative and clinical meetings; supervisory tasks; technical training; establishing standards for reports, protocols, and procedures; participation in and creation of quality programs; review of reports. It is also important to emphasize that, even when these professionals are fully devoted to reading examinations, their work routine encompasses at least four steps^(4)^: pre-examination assessment, which may include interviewing the patient and the referring physician to decide on the appropriateness of the study and the technique chosen; monitoring the execution of the examination to ensure patient safety and image quality; image reading/interpretation, which can include analysis of the patient clinical history and other radiological and laboratory tests; and preparation of the report and possible clarification about the findings to patients and referring physicians.

When estimating the productivity of radiologists, it is inevitable that controversy will arise regarding which form of remuneration would work best. Our results show that radiologists paid through a variable remuneration model, based on the number of reports issued, have higher productivity. The productivity of professionals whose remuneration depends entirely on the number of reports issued is even higher than that of professionals who are paid through a mixed model. However, considering the two extremes, the difference did not exceed 8%. Although productivity-based pay encourages professionals to issue more reports, it can lead to work overload and more diagnostic errors. Studies confirm that an increase in the number of reports issued per shift affects the interpretive accuracy and increases the error rate in those reports^(10,11)^. Sokolovskaya et al.^(10)^ studied radiologists who were asked to interpret imaging examinations at twice their usual speed and found that the number of significant errors increased from 10.0% to 26.6%^(10)^. Other studies have also shown that long workdays with a large volume of examinations to be read reduce the diagnostic accuracy of radiologists^(12,13)^. Diagnostic errors can have several causes other than the time spent on image interpretation, such as work overload, fatigue, excessive interruption, lack of experience, or differences of opinion^(10)^. One important aspect that should be considered is that, in a productivity-based remuneration model, the pay for less complex examinations is the same as that for more challenging examinations. For example, the amount paid for the interpretation of an abdominal CT scan of a previously healthy patient with suspected urolithiasis is the same as that paid for the interpretation of a complex follow-up study of a cancer patient in which detailed measurements and comparisons with prior examinations are required^(2)^. In addition, physicians report greater job satisfaction when their pay or incentives are based on the recognition of their excellence in patient care rather than predominantly on their productivity^(14)^.

Our study showed higher productivity rates among radiologists who worked mainly in the field of telemedicine. That could be explained by the fact that their roles are often, although not exclusively, limited to image interpretation and report writing, allowing for a better quantitative performance. In addition, in this type of work, previous examinations for comparative reports are often inaccessible, possibly leading to a more simplified report which requires less analysis time. There is also a trend toward lower pay for teleradiology reports, which could lead to higher productivity at the risk of commoditization of the service provided, as well as greater exposure of professionals to physical and mental burnout^(15)^.

The fact that the productivity of radiologists working predominantly in public hospitals was lower than was that of those working in the private sector can be explained by poor management and monitoring of productivity in the public system. A study has shown that radiologists are more productive when properly monitored^(16)^. Another factor that is likely to influence this rate is the limited availability of technological resources that have proven to boost productivity, such as integration of the imaging storage, patient medical record, and voice recognition systems, as well as the scanning (digitizing) of physician requests and patient questionnaires^(17)^. In addition to the well-known limitations of the public sector, public hospitals tend to attract a significant number of high-complexity cases that require more analysis time and are often also teaching/ research institutions, which could have an impact on the productive capacity of the clinical staff who end up taking on multiple functions^(4)^.

Although there is a growing trend toward subspecialization among radiologists in Brazil, most of our survey respondents were general radiologists. We find it curious that general radiologists report higher productivity rates than do subspecialists^(6)^. A possible explanation for this finding is that general radiologists tend to write less thorough reports, which would be less time-consuming. Another explanation could be that these professionals are more likely to work at centers that deal with less complex examinations. However, these are only conjectures.

We found that radiologists in Brazil have productivity levels similar to those of radiologists in some other countries^(1,4)^, despite the fact that they may not have the same level of technological support and infrastructure.

## LIMITATIONS AND DIRECTIONS

Although the productivity figures seem to be objective in measuring the work of radiologists, analyzing these data in isolation can lead to an underestimation of other activities that go beyond the mere writing of reports and that are key to a successful radiological practice. For example, participation in professional associations, hospital committees, and conference presentations, at the local and national level, are activities that enhance the practice of the specialty. Developing good relationships with referring physicians and hospital administrators is critical for a successful, long-term career in a particular radiology group and in the medical community as a whole. Teaching (inside or outside the institution) and research activities are also crucial for the prosperity of the specialty and its members^(3)^.

With advances in artificial intelligence, it is expected that the productivity of radiologists will increase as tasks of low cognitive value become automated. As a result, the time spent interfacing with different electronic systems (picture archiving and communication systems, radiology information systems, hospital information systems, etc.) will be reduced, as will that spent identifying and measuring simple imaging findings, and radiologists will be able to spend more time establishing and confirming diagnoses^(18)^. In this context, non-clinical activities should be expanded and encouraged. We have already seen examples of radiology practices that value these tasks, in the form of remuneration, awards, or incentives.

Our study has some limitations. First, we did not take into account the effect of working afterhours and there is evidence that engaging in radiology activities after 6:00 p.m. adversely affects not only professional productivity but also interpretive accuracy and overall quality of life^(13)^. In addition, we did not measure differences between genders and there might be a difference in the perception of what is an acceptable minimum and an acceptable maximum level of productivity^(19)^. Furthermore, our study sample comprised only a small number of thoracic radiologists (n = 15), although that could be a reflection of the radiology market as a whole. Moreover, the low response rate obtained (9.1%) could be considered a limitation, albeit within the expected range for this type of survey^(20)^.

In conclusion, we have shown that, in general, the self-reported productivity of radiologists in Brazil depends on a few factors, such as the place where they live, their remuneration model, and the work environment. We believe that our data can serve as the foundation for and motivate further research that can provide us with increasingly reliable data on the productive capacity of radiologists, going beyond the mere writing of radiological reports to address the relationship between productivity and diagnostic errors.

## Appendix I—Questionnaire on Radiologist Productivity.

**Table:**

1. How old are you?
( ) 20 to 29 ( ) 30 to 39 ( ) 40 to 49 ( ) 50 to 59 ( ) 60+
2. In which Brazilian state do you work?
3. Are you based in the state capital or elsewhere?
( ) Predominantly in the state capital
( ) Predominantly elsewhere
4. What is your predominant work environment?
( ) Private hospital
( ) Public hospital
( ) Private clinic
( ) Teleradiology
5. What is the most common remuneration model through which you are paid?
( ) Productivity-based
( ) Per-shift
( ) Mixed
6. Which imaging method do you employ most often?
( ) Magnetic resonance imaging (MRI)
( ) X-ray
( ) Computed tomography (CT)
( ) Ultrasound (US)
( ) Other
( ) MRI/CT
7. If you marked X-ray, US, CT, or MRI in the question above, what is your predominant subspecialty?
( ) Abdominal/pelvic radiology
( ) Musculoskeletal radiology
( ) Neuroradiology
( ) Thoracic radiology
( ) General radiology
( ) Not applicable
8. What is the MINIMUM (acceptable) number of reports issued in your pre- dominant specialty per 6-hour shift? (note: examination of the upper abdo- men and pelvis = 2 reports). In other words, what would you consider an acceptable number within this time frame?
9. What is the MAXIMUM (acceptable) number of reports issued in your pre- dominant specialty per 6-hour shift? (note: examination of the upper abdo- men and pelvis = 2 reports). In other words, what is the number of reports that, to ensure the quality of the report and the well-being of the radiologist, should not be exceeded?
10. What is your AVERAGE PERSONAL number of reports issued (in your pre- dominant specialty) per 6-hour shift? (note: examination of the upper abdo- men and pelvis = 2 reports).
